# Effect of selenium on markers of risk of pre-eclampsia in UK pregnant women: a randomised, controlled pilot trial

**DOI:** 10.1017/S0007114514000531

**Published:** 2014-04-08

**Authors:** Margaret P. Rayman, Elizabeth Searle, Lynne Kelly, Sigurd Johnsen, Katherine Bodman-Smith, Sarah C. Bath, Jinyuan Mao, Christopher W. G. Redman

**Affiliations:** 1 Department of Nutritional Sciences, School of Biosciences and Medicine, Faculty of Health and Medical Sciences, University of Surrey, GuildfordGU2 7XH, UK; 2 Nuffield Department of Obstetrics and Gynaecology, University of Oxford, OxfordOX3 9DU, UK; 3 Anu Research Centre, Department of Obstetrics and Gynaecology, Cork University Maternity Hospital, Wilton, Cork, Republic of Ireland; 4 Surrey Clinical Research Centre (CRC), Faculty of Health and Medical Sciences, University of Surrey, GuildfordGU2 7XP, UK

**Keywords:** Selenium, Pre-eclampsia, Soluble vascular endothelial growth factor receptor-1, Pregnancy, Selenoprotein P

## Abstract

Pre-eclampsia is a serious hypertensive condition of pregnancy associated with high maternal and fetal morbidity and mortality. Se intake or status has been linked to the occurrence of pre-eclampsia by our own work and that of others. We hypothesised that a small increase in the Se intake of UK pregnant women of inadequate Se status would protect against the risk of pre-eclampsia, as assessed by biomarkers of pre-eclampsia. In a double-blind, placebo-controlled, pilot trial, we randomised 230 primiparous pregnant women to Se (60 μg/d, as Se-enriched yeast) or placebo treatment from 12 to 14 weeks of gestation until delivery. Whole-blood Se concentration was measured at baseline and 35 weeks, and plasma selenoprotein P (SEPP1) concentration at 35 weeks. The primary outcome measure of the present study was serum soluble vascular endothelial growth factor receptor-1 (sFlt-1), an anti-angiogenic factor linked with the risk of pre-eclampsia. Other serum/plasma components related to the risk of pre-eclampsia were also measured. Between 12 and 35 weeks, whole-blood Se concentration increased significantly in the Se-treated group but decreased significantly in the placebo group. At 35 weeks, significantly higher concentrations of whole-blood Se and plasma SEPP1 were observed in the Se-treated group than in the placebo group. In line with our hypothesis, the concentration of sFlt-1 was significantly lower at 35 weeks in the Se-treated group than in the placebo group in participants in the lowest quartile of Se status at baseline (*P*= 0·039). None of the secondary outcome measures was significantly affected by treatment. The present finding that Se supplementation has the potential to reduce the risk of pre-eclampsia in pregnant women of low Se status needs to be validated in an adequately powered trial.

Overall, 10 % of women have high blood pressure during pregnancy and 2–5 % will develop pre-eclampsia, a serious hypertensive condition associated with high maternal and fetal morbidity and mortality^(^ ^1^ ^)^. Furthermore, women who have had pre-eclampsia have a greater risk of developing hypertension, stroke and IHD in later life^(^ ^2^ ^–^ ^4^ ^)^. Numerous strategies to prevent pre-eclampsia have been investigated, but, so far, all have failed to reach effectiveness.

Deficient placentation occurring during the first half of pregnancy is a frequent forerunner of the development of pre-eclampsia^(^ ^5^ ^)^. Shallow trophoblast invasion and inadequate spiral arteriole remodelling result in a placenta that is not adequately perfused^(^ ^5^ ^,^ ^6^ ^)^. Thus, localised areas of ischaemia/reperfusion associated with placental oxidative and endoplasmic reticulum stress are established^(^ ^6^ ^–^ ^9^ ^)^. This results in increased apoptosis and necrosis of the syncytiotrophoblast layer lining the intervillous space^(^ ^10^ ^)^. The oxidatively stressed syncytiotrophoblast responds by the increased release of two anti-angiogenic factors – soluble vascular endothelial growth factor receptor-1 (sVEGFR-1, also called sFlt-1) and soluble endoglin – into the maternal circulation that oppose the actions of vascular endothelial growth factor and placental growth factor (PlGF)^(^ ^11^ ^–^ ^13^ ^)^, leading to endothelial dysfunction, hypertension and proteinuria. Other characteristics of the condition include higher circulating levels of soluble adhesion molecules produced by the activated endothelium^(^ ^14^ ^,^ ^15^ ^)^, and of the potent inflammatory mediator peroxynitrite that causes vasoconstriction, platelet aggregation and thrombus formation^(^ ^16^ ^–^ ^18^ ^)^. In addition, placental microvesicles are released into the maternal circulation where they stimulate a maternal systemic inflammatory response and endothelial activation, the hallmark of pre-eclampsia^(^ ^5^ ^,^ ^19^ ^–^ ^22^ ^)^.

The essential trace mineral Se acting through selenoproteins/selenoenzymes has the capacity to reduce the risk of pre-eclampsia^(^ ^23^ ^–^ ^29^ ^)^. A positive correlation between Se status and the incidence of pre-eclampsia has been shown in an epidemiological study of forty-five countries^(^ ^30^ ^)^. Supplementation of Chinese women deemed to be at risk of pregnancy-induced hypertension (PIH) with Se has been shown to prevent PIH and gestational oedema, two of the signs of pre-eclampsia^(^ ^31^ ^)^. Serum/plasma Se and plasma glutathione peroxidase (GPx) concentrations have been found to be significantly lower in pre-eclamptic than in normal pregnancies^(^ ^32^ ^–^ ^36^ ^)^, while significantly lower levels of the selenoenzymes, GPx and thioredoxin reductase have been found in placentae from pre-eclamptic women than from matched healthy controls^(^ ^8^ ^,^ ^32^ ^,^ ^34^ ^,^ ^37^ ^)^. In a retrospective study in a large Norwegian case–control cohort, women with pre-eclampsia were significantly more likely to carry the A allele of the G105A promoter polymorphism in the anti-inflammatory selenoprotein S (SEPS1)^(^ ^38^ ^)^. In an interesting animal study, a Se-free diet caused a pre-eclampsia-like syndrome in pregnant rats with significantly increased blood pressure, proteinuria and placental oxidative stress and significantly lower pup weight compared with Se-adequate controls^(^ ^39^ ^)^.

In a previous study, we showed that Se status was low in Oxford women with normal (non-pre-eclamptic) pregnancy (median serum Se concentration 48·5 μg/l at 33 weeks)^(^ ^40^ ^)^. In a subsequent case–control study in the same location, we found that the concentration of Se in the toenails (laid down from 3 to 12 months previously) of women with pre-eclampsia was significantly lower than that of matched controls (*P*= 0·001)^(^ ^41^ ^)^. Women in the bottom tertile of toenail Se were 4·4 (95 % CI 1·6, 14·9) times more likely to have pre-eclampsia. Within the pre-eclamptic group, lower Se status was significantly associated (*P*= 0·029) with more severe expression of disease, as measured by delivery before 32 weeks^(^ ^41^ ^)^. Following on from that study, we decided to set up a randomised, placebo-controlled trial, SPRINT (Se in PRegnancy INTervention), to measure the effect of supplementation with dietary levels of Se (i.e. 60 μg/d, the UK-recommended intake for women)^(^ ^42^ ^)^, on the markers of pre-eclamptic risk, inflammation, endothelial and placental function.

Our hypothesis was that a small increase in the intake of Se in pregnant women of inadequate Se status would protect against the risk of pre-eclampsia, as assessed by biomarkers of pre-eclampsia, by increasing Se status and selenoprotein concentration.

The biomarkers that we chose to measure were serum or plasma markers known to be significantly associated with the development of pre-eclampsia. The primary outcome measure of the present study was sFlt-1, a marker of placental dysfunction that blocks the actions of vascular endothelial growth factor and PlGF and impedes the maintenance of endothelial integrity^(^ ^11^ ^,^ ^12^ ^)^. Secondary outcome measures were as follows: PlGF, an angiogenic factor the levels of which are significantly lower in pregnancies where pre-eclampsia subsequently develops^(^ ^43^ ^)^; soluble endoglin, an anti-angiogenic protein that inhibits the formation of capillary tubes and induces vascular permeability and hypertension^(^ ^44^ ^)^; activin A and inhibin A, placental endocrine factors that, when increased, give early warning of pre-eclampsia^(^ ^45^ ^)^; vascular cell adhesion molecule-1 and E-selectin, cell adhesion molecules that are indices of endothelial activation^(^ ^14^ ^,^ ^15^ ^,^ ^46^ ^)^; plasma nitrotyrosine, an indicator of peroxynitrite exposure observed in placental vascular endothelium in pre-eclampsia^(^ ^17^ ^,^ ^18^ ^)^; C-reactive protein (CRP), a measure of inflammation raised in early gestation in advance of pre-eclampsia^(^ ^47^ ^)^; pentraxin-3, an inflammatory marker thought to be more specific to vascular inflammation than CRP^(^ ^48^ ^)^.

## Methods

### Design

The present double-blind, placebo-controlled, two-group study was registered at the International Standard Randomised Controlled Trial Number Registry (registration no. ISRCTN37927591; http://controlled-trials.com/ISRCTN37927591).

### Ethics statement

The present study was conducted according to the guidelines laid down in the Declaration of Helsinki, and all procedures involving human subjects were approved by the Milton Keynes Research Ethics Committee (REC reference no. 08/H0603/46). Written informed consent was obtained from all subjects.

### Participants

We chose to study primiparous women because they have a higher risk of pre-eclampsia. Between 14 July 2009 and 6 June 2011, primiparous women attending the antenatal clinic at the John Radcliffe Hospital, Oxford, UK, for an ultrasound scan at 12 weeks of gestation were invited to join the trial.

### Eligibility criteria

We initially planned to recruit women only in the bottom half of Se status in line with our hypothesis that a treatment effect would only be seen in women of low Se status. However, reviewers of our proposal recommended that we should recruit all women, regardless of Se status, and specify that the primary data analysis would be on women of low Se status. We accepted that modification to our protocol which had the added advantage of avoiding a time lag due to the screening process which would have resulted in our randomising women in pregnancy than we wanted.

The inclusion criteria were first pregnancy and 12–14 weeks of gestation at randomisation.

The exclusion criteria were as follows: under 18 years old; current smokers (who have a lower risk of pre-eclampsia); taking any supplement containing Se; taking thyroid medication; multiple pregnancy; abnormal fetal anomaly scan; chronic proteinuria; heparin treatment; HIV, Hep-B or Hep-C positive; yeast intolerance (supplement contains yeast); inability or refusal to give informed consent (the genetic component of the trial was omitted in those who did not give explicit consent to this aspect of the trial).

### Intervention

Women were randomly allocated to one of two treatment groups: 60 μg/d of Se, as Se-enriched yeast (SelenoPrecise^TM^), or placebo yeast, both supplied by Pharma Nord (60 μg/d is the recommended intake for UK women)^(^ ^42^ ^)^. The treatment period was from randomisation (12 up to a maximum of 14 weeks) until delivery.

A blood sample (10 ml) was taken from all recruits at trial entry. Whole blood was used to measure Se concentration, while the remainder was separated into plasma and serum and banked. If separate, explicit, consent had been given, an additional 5 ml of blood were taken as part of the same procedure for potential genetic analysis. A urine sample was taken for possible future use.

The following information was recorded by the trial midwife: blood pressure; date of the last menstrual period/gestational age; weight; height; haematocrit; ethnicity; family history of pre-eclampsia; chronic illness, e.g. chronic hypertension, diabetes, thyroid disease; current medication; presence of severe morning sickness; alcohol use; vegetarian or not. Women were given a simple FFQ to complete and a further questionnaire about dietary supplements taken.

Women were asked to allow their toenails to grow for 4–6 weeks (for possible later analysis), cut them, place the clippings in the plastic envelope provided to return to the research midwife.

At 20 weeks (when attending for a scan) and 35 weeks, women were again seen at the John Radcliffe Hospital by the research midwife. Blood and urine samples were taken again, separated as appropriate into serum and plasma, and banked for the measurement of different components related to the risk of pre-eclampsia.

Data from the analysis of urine, toenails and FFQ will be the subject of subsequent publications.

### Outcomes

The specified primary outcome measure of the present study was serum concentration of sFlt-1^(^ ^11^ ^,^ ^12^ ^)^. Specified secondary outcome measures were as follows: serum PlGF, soluble endoglin, activin A and inhibin A; plasma VCAM-1, E-selectin, 3-nitrotyrosine and CRP.

An additional outcome introduced because of a concurrent funded study being carried out at the John Radcliffe Hospital was plasma pentraxin-3^(^ ^48^ ^)^.

Se status was measured by whole-blood Se concentration and plasma concentration of selenoprotein P (SEPP1), the carrier of Se in the plasma. Whole-blood concentration of Se, a longer-term measure of Se status than plasma Se, was determined at baseline and 35 weeks. Plasma concentration of SEPP1, a major component of plasma Se and an important parameter of functional Se status, was measured at 35 weeks^(^ ^49^ ^)^.

### Measurement of outcomes

#### Whole-blood selenium

The analysis was carried out at the SAS Trace Element Unit, Southampton General Hospital by dynamic reaction cell-inductively coupled plasma MS on an Elan 6100 DRC plus (SCIEX Perkin-Elmer). Se concentration was measured using methane as the dynamic reaction cell gas to remove the argon dimer background^(^ ^50^ ^)^. Samples were run against a bovine blood calibration curve spiked with Se (Se standard solution 1000 ppm; Fisher Chemical). To increase signal sensitivity, a sample diluent containing 0·5 % butanol^(^ ^51^ ^)^ was used for calibration, testing and quality control. Rhodium was used as the internal standard. A certified reference material (Trace Elements Whole Blood: Seronorm; Sero) assured accuracy: observed values 1·44 (sd 0·25) μmol/l (*n* 42) for lot number 1103129 (target value 1·42 μmol/l) and 3·02 (sd 0·19) μmol/l (*n* 32) for lot number 1003193 (target value 3·29 μmol/l).

#### Selenoprotein P

Frozen plasma samples were shipped to the laboratory of Raymond Burk. Immediately before the analysis, samples were thawed and SEPP1 concentrations were measured using an ELISA as described previously^(^ ^52^ ^)^.

#### Soluble vascular endothelial growth factor receptor-1, placental growth factor and soluble endoglin

Concentrations of sFlt-1, PlGF and soluble endoglin were measured using commercially available Duoset kits for the development of a sandwich ELISA kit (R&D systems), according to the manufacturer's instructions. The intra- and inter-assay CV were 5·2 and 10·5 % for sFlt-1, 5·2 and 8·3 % for PlGF, and 4·9 and 11·6 % for soluble endoglin, respectively.

#### Inhibin A and activin A

The levels of inhibin A and activin A were measured using a two-site ELISA as described previously^(^ ^53^ ^)^. Affinity-purified human activin A was used as the assay standard for the measurement of total activin A. The intra- and inter-assay CV for inhibin A were 5·6 and 12·2 % and for activin A were 6·8 and 10·5 %, respectively.

#### E-selectin and vascular cell adhesion molecule-1

The circulating levels of soluble E-selectin and vascular cell adhesion molecule-1 were measured using Quantikine ELISA Kits (R&D Systems, Inc.), according to the manufacturer's instructions. The intra- and inter-assay CV were 5·8 and 7·9 % for E-selectin and 3·1 and 7·0 % for vascular cell adhesion molecule-1, respectively.

#### 3-Nitrotyrosine

Circulating plasma levels of 3-nitrotyrosine were measured using a Hycult-Biotech ELISA kit (Hycult-Biotech), according to the manufacturer's instructions. The inter- and intra-assay CV were < 15 %.

#### C-reactive protein

Circulating levels of CRP in plasma samples were detected using an in-house, high-sensitivity ELISA adapted from a previously described competition ELISA^(^ ^54^ ^)^ by comparing the direct detection of bound CRP by horseradish-peroxidase-conjugated rabbit anti-human CRP (DakoCytomation) and visualisation using Tetra Methyl Benzidine (Sigma-Aldrich). The intra- and inter-assay CV were 5 and 8 %, respectively.

#### Pentraxin-3

Plasma samples were analysed using a pentraxin 3 ELISA kit (DIESSE Diagnostica Senese SpA), according to the manufacturer's instructions. The between-batch CV was 6·55 % at a pentraxin-3 concentration of 1·92 ng/ml, 7·58 % at 8·48 ng/ml and 8·45 % at 13·34 ng/ml.

### Sample size

We knew that our trial was not powered to look at pre-eclampsia risk *per se*, but believed that we had adequate power to observe a significant difference in our chosen primary end-point of sFlt-1, a recognised marker of pre-eclampsia risk. We calculated sample size using graphical data on sFlt-1 in normal pregnant women^(^ ^11^ ^)^. Taking the standard deviation as 0·2 ng/ml, twenty-nine subjects per group gave us 80 % power to detect a difference of 1·5 ng/ml in the mean sFlt-1 values between the placebo- and Se-treated groups at a two-sided 5 % significance level. As our hypothesis was that an effect of Se supplementation would only be seen in women of inadequate Se status (e.g. in the bottom tertile)^(^ ^41^ ^)^, we increased the sample size to 100 per group to include a sufficient number of such women. To allow for a 15 % dropout, we planned to recruit 115 subjects per group, making a total of 230 subjects. It subsequently came to light that an error had been made, in that the standard deviation of 0·2 ng/ml was actually the standard error, resulting in the present trial being underpowered to detect a difference in the sFlt-1 end-point.

Using the observed difference in means between the Se-treated and placebo groups and the measured standard deviation in both groups, we have now calculated the true power of the present study to achieve two-sided significance at the 5 % level when comparing the means of sFlt-1 in the two arms of the study to be 10 % overall and 36·8 and 54·5 % for women in the bottom tertile and quartile of Se status, respectively. As neither power level is adequate to achieve a significant effect of treatment, we have redefined the present study as a pilot trial.

### Randomisation

Centralised randomisation was performed by the National Perinatal Epidemiology Unit Clinical Trials Unit, University of Oxford. The randomisation schema was created using the subroutine ralloc.ado (version 3.5.2) in Stata software (version 10.1). Treatments were allocated in the ratio of 1:1 using permuted block randomisation with variable block sizes of 2, 4 and 6 (seed used: 221010). Block sizes were allocated proportional to the elements of Pascal's triangle.

A third party, Pharma Nord, who were not involved in any way in the recruitment of participants for the trial, provided the treatment tablets. Pharma Nord was notified of the allocation sequence by an independent statistician at the National Perinatal Epidemiology Unit, and packed eight blister strips of twenty-eight tablets into plain white boxes.

The pharmacist at the John Radcliffe Hospital held and dispensed the boxes, each of which bore a label with a unique pack identification number. Each blister pack within each box also bore the randomisation number. The packs were collected by the research midwife from pharmacy as required. A log was kept which, on dispensing the packs, was signed by two people, the research midwife and pharmacist or nurse prescriber. The pack label was also signed before giving the trial box to each participant.

#### Blinding

Apart from the independent statistician at the National Perinatal Epidemiology Unit who generated the sequence, all study personnel and the participants were blinded to allocation.

### Assessment of compliance

Compliance was assessed by tablet count. Stamped, addressed envelopes were provided to participants who were instructed to return empty/partially used/unused blister packs by post to the research midwife who recorded the number of tablets taken. The midwife contacted those who failed to return the packs from the previous 4 weeks within 10 d of the expected return date by telephone. To assess compliance, the total number of study tablets reported as having been taken was expressed as a percentage of the number of days between the study consent date and the date of giving birth. Participants were deemed to be compliant if they took 80 % of their study tablets.

### Rationale for an approach to data analysis

We observed a large and significant reduction (12 %) in whole-blood Se concentration from 12 to 35 weeks in the placebo group in the present study, resulting, at least in part, from the increase in plasma volume that occurs over the course of normal pregnancy^(^ ^55^ ^)^. We found that we could mitigate this dilution effect by correcting Se concentration by haematocrit, which reduced the decline in the concentration of Se in the placebo group to 7 %. Therefore, this adjustment was made in some of the analyses as explained below.

As the earlier study on which the present pilot trial was based^(^ ^41^ ^)^ showed a significantly higher incidence of pre-eclampsia in women in the bottom tertile of toenail Se than in the rest, analyses in subgroups with low baseline Se status were the pre-specified primary analyses. As the earlier study and this trial used different matrices (toenail Se and whole-blood Se, respectively) and toenail cuttings reflect Se status before pregnancy, we endeavoured to determine the equivalent baseline whole-blood Se cut-off point in pregnant women to that used for toenail Se (bottom tertile cut-off point 0·547 mg/kg) in the previous study. Using the data from Behne *et al.* ^(^ ^56^ ^)^ and taking the density of whole blood to be 1·06 kg/l, we calculated the equivalent whole-blood Se cut-off point to be 106·5 μg/l. As pregnancy causes an expansion of blood volume from the non-pregnant state, the equivalent whole-blood Se cut-off value would be 95·1 μg/l, assuming that the increase was the same as that observed in plasma volume at 12 weeks of gestation, i.e. 12 %^(^ ^57^ ^)^. This value represents a cut-off between the bottom third and the bottom quarter of baseline whole-blood Se. Therefore, we examined the treatment effect in both the bottom quartile and the bottom tertile of Se status by blood Se concentration at baseline.

### Statistical analysis

#### Intention-to-treat analysis

None of the dependent variables was normally distributed, nor was baseline Se; therefore, they were log-transformed to facilitate parametric testing. Thus, medians (ranges) or geometric means and their 95 % CI, computed by back transformation of log values, are reported. All analyses of continuous data used one observation per subject in a general linear model (SAS PROC MIXED), with treatment (Se or placebo) as the independent variable. Additional covariates were included as appropriate. These covariates were log baseline blood Se (adjusted or unadjusted for baseline haematocrit) and log baseline haematocrit. The treatment effect was assessed using the LSMEANS and PDIFF options of this SAS statistical procedure.

The pre-specified primary data analysis of this study was carried out on the subgroup of women with low Se levels at recruitment (the lowest third or the lowest quarter). Subgroups with low Se status (quartiles and tertiles) were defined as blood Se concentration at baseline divided by haematocrit concentration at baseline.

#### Sensitivity analysis

##### Soluble vascular endothelial growth factor receptor-1 by compliance

The analysis was repeated including only those women who had taken 80 % or more of their trial tablets.

#### Exploratory subgroup analyses

##### Soluble vascular endothelial growth factor receptor-1 by Se quartile at baseline

Quartiles of baseline blood Se and treatment were simultaneously contrasted to determine whether a trend could be observed across quartiles. The quartile was an additional categorical independent variable in the model and the contrast was assessed using the ESTIMATE option of the PROC MIXED procedure.

##### Pre-eclampsia and pregnancy-induced hypertension

Pre-eclampsia and PIH were not pre-specified outcomes. PIH is defined as new hypertension appearing for the first time after 20 weeks of pregnancy. Hypertension is defined as diastolic blood pressure ≥ 90 mmHg on two occasions ( ≥ 4 h apart). Pre-eclampsia is defined as PIH and new-onset proteinuria after 20 weeks of pregnancy ( ≥ 300 mg/24 h or ≥ 2+ dipstick midstream specimen of urine/catheter specimen of urine). A protein:creatinine ratio of >30 mg/mmol of creatinine was accepted as confirmation of proteinuria. These criteria are an extension of those adopted by the International Society for the Study of Hypertension in Pregnancy^(^ ^58^ ^)^. Women were dichotomised according to whether they had either pre-eclampsia/PIH, or neither. The relationship with study treatment (Se or placebo) was then analysed by logistic regression.

#### Associations with baseline selenium status

The correlation of whole-blood Se (log-transformed and haematocrit-corrected) at baseline with other parameters was investigated using appropriate statistical tests.

## Results

### Participants

**Fig. 1:**
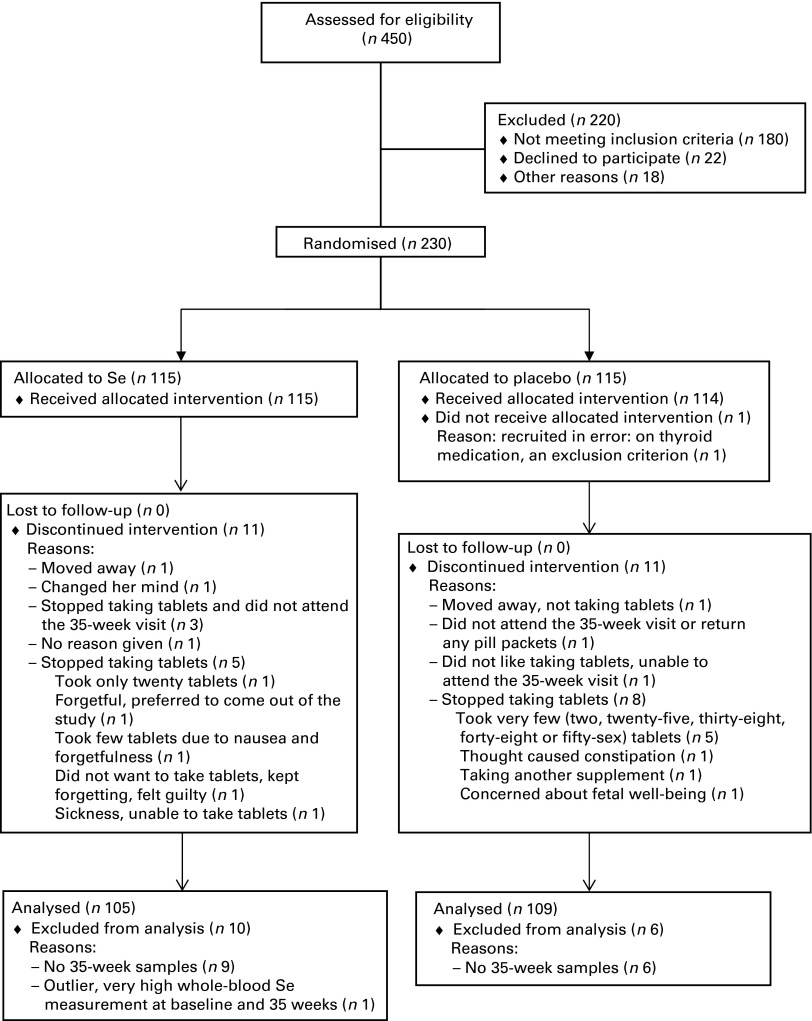
Participant flow through the study.

A total of 230 participants were recruited between July 2009 and June 2011. The mean gestational age at recruitment was 12·3 weeks. One participant was recruited at 16 weeks; she was included in the data analysis. In each treatment group, eleven participants discontinued the treatment. Figure 1 shows the participant flow through the study.

The baseline characteristics of the participants are shown in Table 1 from which it can be seen that there were no significant differences between the groups at baseline.

**Table 1:** Baseline characteristics of the 229 participants, overall and by treatment group (Mean values and standard deviations; numbers and percentages; medians and ranges)

	Overall (*n* 229)	Se-treated group (*n* 115)	Placebo group (*n* 114)	
	Mean	sd	Mean	sd	Mean	sd	*P* *
Age (years)	30·73	4·18	30·35	4·55	31·11	3·75	0·166
Gestational age at recruitment (weeks)	12·30	0·88	12·26	0·85	12·34	0·91	0·500
Age of mother at the time of leaving education (years)	20·79	2·97	20·59	2·87	21·00	3·07	0·296
Ethnicity (Caucasian)							0·124
*n*	213	104	109	
%	93·0	90·4	95·6	
BMI (kg/m^2^)	24·59	3·99	24·77	4·11	24·41	3·88	0·499
Underweight, BMI < 18·5 kg/m^2^							0·361†
*n*	2	0	2	
%	0·9	0	1·8	
Normal body weight, BMI 18·5–24·9 kg/m^2^							
*n*	136	65	71	
%	59·4	56·5	62·3	
Overweight, BMI 25–29·9 kg/m^2^							
*n*	70	38	32	
%	30·6	33·0	28·1	
Obese, BMI ≥ 30 kg/m^2^							
*n*	21	12	9	
%	9·2	10·4	7·9	
Non-smoker							0·098
*n*	156	83	73	
%	68·1	72·2	64·0	
Stopped smoking before conception							
*n*	59	23	6	
%	25·8	20·0	31·6	
Stopped smoking when pregnant							
*n*	14	9	5	
%	6·1	7·8	4·4	
Consumes alcohol							0·669
*n*	134	66	68	
%	58·8	57·4	60·2	
Se status: whole-blood Se concentration (μmol/l)							0·352
Median	1·31	1·31	1·32	
Range	0·84–3·33	0·84–3·33	0·84–2·19	
Pentraxin 3	1·09	0·42	1·09	0·40	1·10	0·44	0·858

* *P* value for a comparison between the Se-treated and placebo groups.

† *P* value for the distribution of BMI categories between the Se-treated and placebo groups.

The mean gestational age at delivery was 39·65 (95 % CI 39·45, 39·86) weeks overall, 39·75 (95 % CI 39·50, 40·00) weeks in the Se-treated group and 39·56 (95 % CI 39·24, 39·88) weeks in the placebo group, with no significant difference being observed between the Se-treated and placebo groups.

### Selenium status

**Fig. 2:**
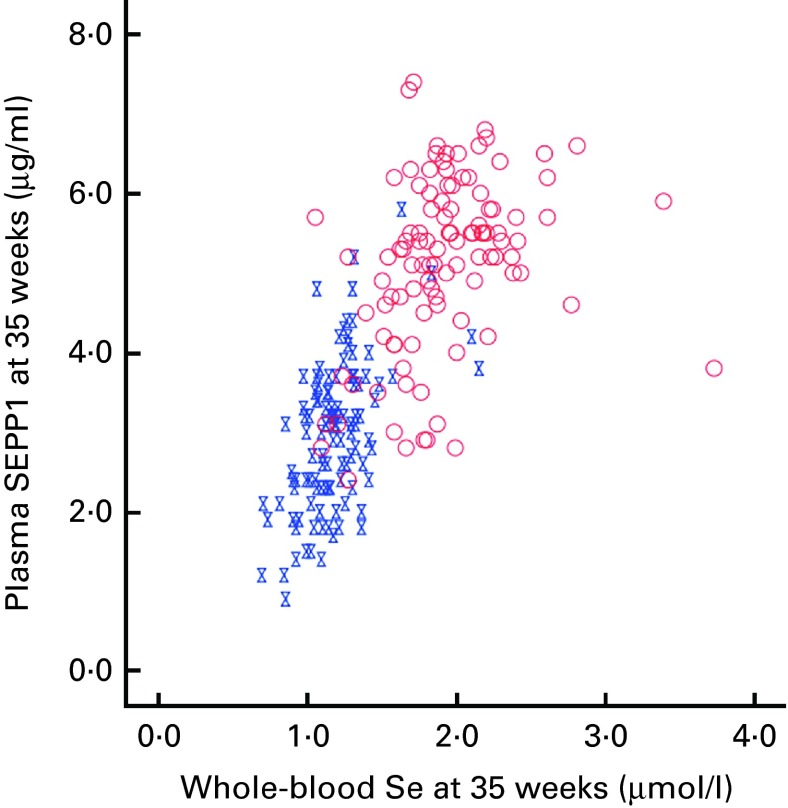
Selenoprotein P (SEPP1) *v*. whole-blood selenium concentration at 35 weeks. 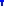, Placebo; 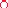, selenium. (A colour version of this figure can be found online at http://www.journals.cambridge.org/bjn)

Blood was successfully obtained from the 230 participants at baseline, though one was subsequently found to be ineligible for the trial, and from 215 participants at the 35-week visit. The overall median whole-blood Se concentration at baseline was 1·31 (range 0·84–3·33) μmol/l (Table 1). By 35 weeks, whole-blood Se concentration had increased significantly in the Se-treated group (see Table 2), whereas there was a significant reduction (12 %) in the placebo group. Whole-blood Se concentration and plasma SEPP1 concentration were significantly higher at 35 weeks in the Se-treated group than in the placebo group (Table 2). Figure 2 shows the difference in the effect of Se *v.* placebo treatment on plasma SEPP1 concentration and whole-blood Se concentration at 35 weeks.

**Table 2:** Effect of selenium supplementation on the parameters of selenium status (Median values and ranges)

	Placebo	Se	
Parameter of Se status	Median	Range	Median	Range	*P* *
Whole-blood Se concentration (μmol/l)					
Baseline (*n* 230)	1·32	0·84–2·19	1·31	0·95–3·33	0·3524
35 weeks (*n* 215)	1·16	0·69–2·15	1·87	1·05–3·73	< 0·0001
*P* (baseline *v.* 35 weeks)	< 0·0001		< 0·0001		
Plasma SEPP1 concentration (μg/ml)					
35 weeks (*n* 215)	3·00	0·90–5·80	5·30	2·40–7·40	< 0·0001

SEPP1, selenoprotein P.

* *P* value for a comparison between the placebo and Se-treated groups at 35 weeks.

### Compliance with treatment

Of the participants, 22 % in the Se-treated group and 23 % in the placebo group were non-compliant, i.e. took less than 80 % of the trial tablets. However, 28 % of the participants failed to return some or all of the empty packets, though many claimed to have taken the tablets; compliance may therefore have been somewhat better than the estimates suggest.

In the placebo group, one participant took a pregnancy supplement containing 30 μg Se sporadically from 16 to 20 weeks of pregnancy along with the trial tablets. She was included in the data analysis.

### Effect of treatment: primary outcome measure (serum soluble vascular endothelial growth factor receptor-1)

The intention-to-treat analysis included all participants (apart from one clear outlier) for whom we had an sFlt-1 measurement, regardless of compliance, i.e. 105 participants in the Se-treated group and 109 in the placebo group (see Fig. 1). Table 3 shows the effect of Se *v.* placebo supplementation on sFlt-1 concentration at 35 weeks in participants in the bottom quartile and tertile of whole-blood Se at baseline (primary data analysis of the present study) and in the whole dataset. Because the ratio of sFlt-1:PlGF is often taken to be a more sensitive indicator of the risk of pre-eclampsia as the fall in the concentration of PlGF amplifies the concurrent rise in the concentration of sFlt-1^(^ ^12^ ^,^ ^59^ ^)^, the data for PlGF and the ratio of sFlt-1:PlGF are also presented. In participants in the lowest quartile of Se status at baseline, treatment with Se significantly lowered the concentration of sFlt-1 (*P*= 0·039) and halved the ratio of sFlt-1:PlGF, though the latter effect did not quite reach significance (*P*= 0·066); however, in all participants taken together, Se treatment had no effect on sFlt-1 concentration, PlGF concentration or sFlt-1:PlGF ratio.

**Table 3:** Effect of selenium supplementation *v.* placebo on soluble vascular endothelial growth factor receptor-1 (sFlt-1) concentration, placental growth factor (PlGF) concentration and the ratio of sFlt-1:PlGF at 35 weeks in participants in the bottom quartile* (*n* 50) and tertile* (*n* 67) of selenium status at baseline and in all participants† ‡ (*n* 214) (Mean values and 95 % confidence intervals)

Outcome measure	Se status group at baseline	Se treatment (geometric mean)	Placebo(geometric mean)	Se:placebo ratio	*P* (Se treatment *v*. placebo)
Mean	95 % CI
sFlt-1 (ng/ml)	Bottom quartile	5·22	7·49	0·70	0·49, 0·98	0·039
	Bottom tertile	5·79	7·55	0·77	0·56, 1·06	0·104
	All participants	6·79	7·18	0·95	0·80, 1·12	0·511
PlGF (ng/ml)	Bottom quartile	261	193	0·97	0·78, 1·21	0·185
	Bottom tertile	241	210	1·15	0·77, 1·71	0·494
	All participants	233	240	0·97	0·78, 1·21	0·801
sFlt-1:PlGF	Bottom quartile	0·020	0·039	0·51	0·25, 1·05	0·066
	Bottom tertile	0·024	0·036	0·67	0·35, 1·27	0·215
	All participants	0·029	0·030	0·97	0·70, 1·36	0·875

* For division into quartiles/tertiles, baseline Se concentration was adjusted for baseline haematocrit.

† In all participants, adjustment for blood Se and haematocrit covariates at baseline and for haematocrit at 35 weeks made no substantial difference to the results.

‡ Analysis on all participants was not part of the primary analysis.

### Effect of treatment: secondary outcome measures

The effect of Se and placebo treatments on secondary outcome measures is shown in Table 4. None of the parameters was significantly affected by Se treatment either in the participants in the bottom tertile or quartile of Se status or in the whole group.

**Table 4:** Effect of selenium supplementation *v.* placebo on secondary outcome measures at 35 weeks in participants in the bottom quartile* and tertile* of selenium status at baseline and in all participants† ‡ (Mean values and 95 % confidence intervals)

Outcome measure	Se status group at baseline	*n*	Se treatment (geometric mean)	Placebo (geometric mean)	Se:placebo ratio	*P* (Se treatment *v.* placebo)
Mean	95 % CI
Soluble endoglin (ng/ml)	Bottom quartile	50	62·32	71·89	0·87	0·69, 1·10	0·227
	Bottom tertile	67	69·08	68·71	1·01	0·78, 1·30	0·967
	All participants	213	64·88	64·72	1·00	0·87, 1·16	0·973
Activin (ng/ml)	Bottom quartile	50	15·36	16·73	0·92	0·66, 1·28	0·606
	Bottom tertile	67	16·71	17·29	0·97	0·72, 1·31	0·821
	All participants	214	17·08	18·38	0·93	0·80, 1·08	0·347
Inhibin (ng/ml)	Bottom quartile	50	1185	1247	0·95	0·69, 1·31	0·749
	Bottom tertile	67	1304	1323	0·99	0·74, 1·30	0·916
	All participants	214	1252	1201	1·04	0·90, 1·21	0·578
VCAM-1 (ng/ml)	Bottom quartile	50	600	649	0·92	0·80, 1·06	0·256
	Bottom tertile	67	611	641	0·95	0·84, 1·08	0·427
	All participants	214	602	618	0·97	0·91, 1·04	0·407
E-selectin (ng/ml)	Bottom quartile	50	31·93	30·49	1·05	0·79, 1·01	0·741
	Bottom tertile	67	32·33	30·93	1·05	0·82, 1·33	0·715
	All participants	214	27·37	30·71	0·89	0·79, 1·39	0·070
CRP (ng/ml)	Bottom quartile	50	3735	2828	1·32	0·87, 1·84	0·448
	Bottom tertile	67	3044	2269	1·34	0·67, 2·69	0·402
	All participants	214	3204	2538	1·26	0·64, 2·75	0·222
Pentraxin 3 (ng/ml)	Bottom quartile	50	1·41	1·49	0·94	0·92, 1·18	0·592
	Bottom tertile	67	1·48	1·63	0·90	0·71, 1·15	0·411
	All participants	214	1·62	1·55	1·04	0·76, 1·18	0·501
Nitrotyrosine (μmol/l)	Bottom quartile	11	2·46	0·81	3·05	0·04, 218·8	0·453
	Bottom tertile	15	2·56	0·85	3·02	0·14, 66·29	0·570
	All participants	49	0·96	0·82	1·18	0·31, 4·52	0·805

VCAM-1, vascular cell adhesion molecule-1; CRP, C-reactive protein.

* For division into quartiles/tertiles, baseline Se concentration was adjusted for baseline haematocrit.

† In all participants, adjustment for blood Se and haematocrit covariates at baseline and for haematocrit at 35 weeks made no substantial difference to the results.

‡ Analysis on all participants was not part of the primary analysis.

### Subgroup analyses (exploratory)

#### Soluble vascular endothelial growth factor receptor-1 by compliance

Reanalysis of the effect of treatment on sFlt-1 concentration when only participants who reported taking 80 % of the trial tablets were included made no difference to the result (data not shown).

#### Soluble vascular endothelial growth factor receptor-1 by quartile of baseline selenium status

Given the significant effect of Se treatment on sFlt-1 concentration in women in the lowest quartile of Se status at baseline, we decided to determine whether the effect showed a trend by quartile. The ratio of sFlt-1 means shown in Table 5 illustrates that, rather than a trend, treatment with Se appears to reduce sFlt-1 concentration only in participants in the bottom quartile of Se status at baseline, with no effect being observed in those in the other quartiles. There is a near-significant difference between the effect in the bottom quartile and the rest (ratio for the lowest quartile compared with the other quartiles: 0·67, 95 % CI 0·45, 1·00; *P*= 0·051).

**Table 5:** Results for soluble vascular endothelial growth factor receptor-1 by quartile of whole-blood selenium concentration at baseline (Mean values and 95 % confidence intervals)

Data subset by quartile of Se at baseline*	*n*	Se treatment, geometric mean (ng/ml)	Placebo geometric mean (ng/ml)	Se:placebo ratio	*P* (Se treatment *v.* placebo)
Mean	95 % CI
1st (bottom) quartile	50	5·22	7·49	0·70	0·49, 0·98	0·039
2nd quartile	51	7·53	7·46	1·01	0·70, 1·45	0·962
3rd quartile	51	7·35	6·45	1·14	0·80, 1·62	0·457
4th quartile	51	7·32	7·52	0·97	0·69, 1·38	0·880

* Division into quartiles by baseline Se status corrected for haematocrit.

#### Pre-eclampsia and pregnancy-induced hypertension

Neither pre-eclampsia nor PIH was a designated outcome because of the known lack of power; there were eleven cases of pre-eclampsia (eight in the placebo group and three in the Se-treated group), and nine cases of PIH (six in the placebo group and three in the Se-treated group). As expected, the effect of Se treatment on the incidence of either outcome failed to reach significance (data not shown). However, when the two outcomes were combined on the basis that there may be some continuity between the conditions^(^ ^60^ ^–^ ^63^ ^)^, and when the analyses were adjusted for baseline Se concentration and haematocrit, Se treatment significantly reduced the odds of having either pre-eclampsia or PIH in all participants (OR 0·350, 95 % CI 0·126, 0·974; *P*= 0·044) and with near significance in those in the bottom third of Se status at baseline (Table 6), with large treatment effects.

**Table 6:** Effect of selenium treatment *v*. placebo on the risk of pre-eclampsia (PE) and pregnancy-induced hypertension (PIH) combined, by treatment group, in all participants and in those in the bottom tertile* and quartile* (Odds ratios and 95 % confidence intervals)

		Subjects with PE/PIH (*n*)	PE/PIH combined
Data subset	*n*	Se-treated group	Placebo group	OR	95 % CI	*P* †
All participants	228	6	14	0·42	0·16, 1·09	0·075
All participants‡	215	5	14	0·35‡	0·13, 0·97	0·044
Bottom Se tertile	71	1	8	0·17	0·03, 1·07	0·059
Bottom Se quartile	53	0	4	0·12	0·01, 2·40	0·163

* Division into tertiles/quartiles by baseline Se concentration corrected for haematocrit.

† χ^2^ test.

‡ Adjusted for baseline Se concentration and baseline haematocrit.

### Associations with selenium at baseline

At baseline (12 weeks), with adjustment for haematocrit, whole-blood Se was negatively correlated with BMI (Pearson's correlation (*r*) − 0·222; *P*= 0·001); there were no other significant correlations.

### Adverse events

Overall two adverse events were reported: one participant had a rash on her legs below the knees, deemed to be mild, and possibly related to the study treatment. Another participant had spontaneous torsion of her right ovary that required right oophorectomy, a rare complication of pregnancy, not connected with the study supplement.

## Discussion

### Effect on selenium status

We used whole blood to measure Se concentration as it gives a longer-term measure than does plasma Se. Se supplementation from 12 to 35 weeks increased whole-blood Se concentration by 43 % in the Se-treated group, while there was a significant fall of 12 % in the placebo group (Table 2). Other studies that measured whole-blood Se in pregnancy have also found a substantial fall in its concentration with increasing gestational age^(^ ^64^ ^,^ ^65^ ^)^. Apart from plasma volume expansion, an additional cause of a fall in blood Se concentration throughout pregnancy may be the transfer of Se to the fetus by SEPP1, a major component of plasma Se that transports Se to other tissues^(^ ^49^ ^)^. This has been suggested by a recent study by Burk *et al.* ^(^ ^66^ ^)^ in pregnant mice that identified two mechanisms of Se transfer; from early to mid-gestation, plasma GPx and SEPP1 were transported via the uterine fluid, probably by pinocytosis, whereas in the latter half of gestation, placental transfer of maternal SEPP1 occurred through apoE receptor 2. Even in mice with normal Se status, maternal plasma SEPP1 concentration fell rather dramatically in late pregnancy with a sharp rebound the day after delivery. Therefore, pregnancy may be putting similar pressure on the Se stores of UK pregnant women with marginal Se status. Women supplemented with Se had at least partial correction of their Se deficiency, raising the SEPP1 concentration, though most participants did not reach the maximum value (see Fig. 2).

### Per-protocol analyses

sFlt-1 was the primary outcome measure of the present study, and our hypothesis was that there would only be an effect of Se supplementation in pregnant women of inadequate Se status. Owing to an initial statistical error, the trial was powered only at 36·8 and 54·5 % to observe a significant difference between sFlt-1 means in the two arms of the study in women in the lowest third and lowest quarter of Se status, respectively. Therefore, it is important that we found a significant difference in participants in the lowest quartile of Se status at baseline, in whom Se treatment lowered the concentration of sFlt-1 by 30 % (ratio of means 0·70, 95 % CI 0·49, 0·98; *P*= 0·039; Table 3). When we examined the effect of Se supplementation on the ratio of sFlt-1:PlGF, a more sensitive indicator of risk of pre-eclampsia^(^ ^12^ ^,^ ^59^ ^)^, we found that it had decreased by 49 % in the bottom quartile, though the effect did not quite reach significance (*P*= 0·066). We observed no significant effect of treatment on any of the secondary outcome measures of the present study, perhaps because of the lack of power, so these will not be discussed further.

What are the mechanisms by which treatment with Se could cause a fall in sFlt-1 concentration, a primary marker of pre-eclampsia risk? Selenoproteins are able to reduce oxidative stress, endoplasmic reticulum stress and inflammation, characteristics of pre-eclampsia, while protecting endothelial cells and up-regulating the expression of the protective haem oxygenase 1 (HO-1)^(^ ^23^ ^–^ ^26^ ^,^ ^28^ ^,^ ^29^ ^,^ ^67^ ^,^ ^68^ ^)^. Se supplementation was found to decrease NF-κB activation and down-regulate the expression of inflammatory genes^(^ ^67^ ^,^ ^68^ ^)^. Both GPx and thioredoxin reductase have been shown to protect endothelial cells from oxidants including oxidised LDL^(^ ^25^ ^,^ ^26^ ^)^. Thioredoxin reductase has been shown to up-regulate the expression of HO-1 by a number of pathways during a pro-oxidant challenge, resulting in the reduced expression of pro-inflammatory genes^(^ ^28^ ^,^ ^29^ ^)^; HO-1 has antioxidant effects and is linked to successful placentation, inhibition of sFlt-1 release, uterine quiescence, placental haemodynamic control and the regulation of apoptotic and inflammatory cascades in trophoblast cells^(^ ^69^ ^–^ ^72^ ^)^. SEPP1, which is recruited to the endothelium in the areas of inflammation, scavenges the powerful inflammatory agent peroxynitrite, thereby reducing oxidative stress and inflammation and shielding endothelial membranes from its attack^(^ ^16^ ^,^ ^73^ ^)^. Gene expression of *SEPS1* is activated by endoplasmic reticulum stress, and binding sites exist for NF-κB in the human *SEPS1* promoter; thus, SEPS1 plays a key role in the control of the inflammatory response^(^ ^27^ ^)^.

The effects of Se outlined above can mostly be attributed to selenoproteins; therefore, it is entirely understandable that we only observed an effect in the bottom quartile of Se status in the present study and in the bottom tertile in our earlier study^(^ ^41^ ^)^. Above a whole-blood Se concentration of 1·3 μmol/l, plasma GPx activity is maximised^(^ ^74^ ^)^. The mean whole-blood Se concentration in trial participants at baseline was 1·31 μmol/l, suggesting that approximately 50 % would not have had maximal plasma GPx activity. If the effects that we observed are attributable to GPx, then only a subgroup of women in the present study could have benefited from Se supplementation, as observed by our group. In contrast to plasma GPx, the concentration of SEPP1 does not plateau until a plasma Se concentration of 1·6 μmol/l is reached^(^ ^75^ ^)^. The results suggest that in whole blood, SEPP1 concentration may plateau at about 1·8 μmol/l (Fig. 2), which accords with the findings in plasma. Supplementation with Se from 12 to 35 weeks was insufficient to raise the concentration of SEPP1 to that plateau level in the majority of women in the Se-treatment group (Fig. 2). Therefore, if beneficial effects are related to the effect of SEPP1, a higher dose or an intervention earlier than 12 weeks of gestation would have been required to observe it.

### Exploratory analyses

In addition to the pre-specified analyses described above, we carried out hypothesis-generating exploratory analyses in order to have some further insight into the effect of Se supplementation.

When we analysed sFlt-1 concentration by quartiles to determine whether a trend could be detected, we observed a reduction in the concentration of sFlt-1 only in participants in the bottom quartile of Se status at baseline, with those in the other quartiles showing no effect (Table 5). Thus, there was a near-significant difference between the effect in the bottom quartile and the rest (*P*= 0·051), suggesting a threshold effect that affected only participants in the bottom quartile of Se status.

We had very small numbers of participants affected by pre-eclampsia^(^ ^11^ ^)^ and PIH^(^ ^9^ ^)^, so we combined the two outcomes on the basis that there may be some continuity between the conditions^(^ ^62^ ^,^ ^63^ ^)^. Se treatment significantly reduced the odds of the combination (pre-eclampsia/PIH) in all participants (OR 0·35, 95 % CI 0·13, 0·97; *P*= 0·044) and with near significance (*P*= 0·059) in those in the bottom third of Se status at baseline, with large treatment effects (eight women developed pre-eclampsia/PIH in the placebo group compared with one in the Se-treated group) (Table 6). Although pre-eclampsia and PIH are not synonymous, the distinction between the two is not always completely clear; 15–45 % of women with PIH will eventually develop pre-eclampsia^(^ ^62^ ^,^ ^63^ ^)^. The earlier the time of presentation, the more likely is PIH to be a harbinger of pre-eclampsia^(^ ^60^ ^)^. If PIH is associated with some other feature of pre-eclampsia than proteinuria, such as hyperuricaemia or intra-uterine growth retardation, it will be now recognised as a possible atypical variant of pre-eclampsia^(^ ^61^ ^)^.

### Baseline associations

The negative correlation (*r* − 0·222; *P*= 0·001) between baseline whole-blood Se and BMI is in line with other findings in the literature on the relationship between serum/plasma Se and BMI in non-pregnant groups^(^ ^76^ ^–^ ^78^ ^)^.

### Trial limitations

The major limitation of the present study was in having very limited power (only 54·5 % in the bottom quartile and 36·8 % in the bottom tertile of participants) to observe a difference in our primary endpoint of sFlt-1.

Intervention at 12 weeks of gestation may have been later than ideal. Our earlier study^(^ ^41^ ^)^ that found a significant difference in Se status between pre-eclamptic and matched control pregnant women used toenail clippings that probably reflected Se status before conception, as toenails are laid down up to 12 months before clipping. We were always aware that if the effect of Se supplementation was primarily in the early stages of gestation, we would not be able to observe it with the present protocol. A number of processes that occur during the periconceptual period might benefit from protection by selenoproteins. Indeed, periconception ‘multivitamin’ (often including Se) use has been shown to reduce the risk of pre-eclampsia^(^ ^79^ ^)^.

We have observed an effect in the bottom quartile (upper cut-off value 1·18 μmol/l in whole blood) of Se status in UK pregnant women. This might roughly equate to 1·07 μmol/l (84·5 μg/l) in serum/plasma. These results may therefore only be generalisable to other population groups of similar Se status such as that likely to be found in other countries of Europe. Vanderlelie & Perkins^(^ ^30^ ^)^ found a significant reduction (*P*= 0·0007) in the incidence of pre-eclampsia in countries with a reported serum/plasma Se concentration ≥ 95 μg/l (1·2 μmol/l). Our trial population falls within that range.

### Suggestions for future work

To date, this is the only study to have investigated the effect of Se supplementation in a population with low Se status that examined an outcome related to the risk of pre-eclampsia. Given the present findings of significantly lower concentrations of sFlt-1 in women in the bottom quartile of Se status supplemented with Se, and the suggestion that Se supplementation may lower the incidence of PIH+pre-eclampsia, the trial should be repeated, properly powered for the outcome of pre-eclampsia (and PIH), in a population with a Se status as low, or lower, than that of the present study. Ideally, recruitment would start earlier than 12 weeks. On the basis of our inability to optimise SEPP1 during the course of the trial, we would recommend a higher, though safe, dose of Se-enriched yeast, i.e. 100 μg/d^(^ ^80^ ^)^. A major difficulty in conducting such a trial in a Western country is recruiting sufficient women who are not taking a pregnancy supplement; such supplements are increasingly available at relatively low cost in supermarket chains.

Despite these difficulties, the potential benefits to be gained by conducting a properly powered trial would make such a demanding exercise worthwhile.

## References

[1] Duley L (2009) The global impact of pre-eclampsia and eclampsia. Semin Perinatol 33, 130–137 19464502 10.1053/j.semperi.2009.02.010

[2] Wilson BJ, Watson MS, Prescott GJ, et al. (2003) Hypertensive diseases of pregnancy and risk of hypertension and stroke in later life: results from cohort study. BMJ 326, 845–851 12702615 10.1136/bmj.326.7394.845 PMC153466

[3] Bellamy L, Casas JP, Hingorani AD, et al. (2007) Pre-eclampsia and risk of cardiovascular disease and cancer in later life: systematic review and meta-analysis. BMJ 335, 974–985 17975258 10.1136/bmj.39335.385301.BE PMC2072042

[4] Levine RJ, Vatten LJ, Horowitz GL, et al. (2009) Pre-eclampsia, soluble fms-like tyrosine kinase 1, and the risk of reduced thyroid function: nested case–control and population based study. BMJ 339, b4336 19920004 10.1136/bmj.b4336 PMC2778749

[5] Redman CW & Sargent IL (2000) Placental debris, oxidative stress and pre-eclampsia. Placenta 21, 597–602 10985960 10.1053/plac.2000.0560

[6] Hubel CA (1999) Oxidative stress in the pathogenesis of preeclampsia. Proc Soc Exp Biol Med 222, 222–235 10601881 10.1177/153537029922200305

[7] Burton GJ & Jauniaux E (2004) Placental oxidative stress: from miscarriage to preeclampsia. J Soc Gynecol Investig 11, 342–352 10.1016/j.jsgi.2004.03.003 15350246

[8] Vanderlelie J, Venardos K, Clifton VL, et al. (2005) Increased biological oxidation and reduced anti-oxidant enzyme activity in pre-eclamptic placentae. Placenta 26, 53–58 15664411 10.1016/j.placenta.2004.04.002

[9] Burton GJ, Yung HW, Cindrova-Davies T, et al. (2009) Placental endoplasmic reticulum stress and oxidative stress in the pathophysiology of unexplained intrauterine growth restriction and early onset preeclampsia. Placenta 30, S43–S48 19081132 10.1016/j.placenta.2008.11.003 PMC2684656

[10] Hung TH, Skepper JN, Charnock-Jones DS, et al. (2002) Hypoxia–reoxygenation: a potent inducer of apoptotic changes in the human placenta and possible etiological factor in preeclampsia. Circ Res 90, 1274–1281 12089065 10.1161/01.res.0000024411.22110.aa

[11] Maynard SE, Min JY, Merchan J, et al. (2003) Excess placental soluble fms-like tyrosine kinase 1 (sFlt1) may contribute to endothelial dysfunction, hypertension, and proteinuria in preeclampsia. J Clin Invest 111, 649–658 12618519 10.1172/JCI17189 PMC151901

[12] Levine RJ, Maynard SE, Qian C, et al. (2004) Circulating angiogenic factors and the risk of preeclampsia. N Engl J Med 350, 672–683 14764923 10.1056/NEJMoa031884

[13] Venkatesha S, Toporsian M, Lam C, et al. (2006) Soluble endoglin contributes to the pathogenesis of preeclampsia. Nat Med 12, 642–649 16751767 10.1038/nm1429

[14] Daniel Y, Kupferminc MJ, Baram A, et al. (1999) A selective increase in plasma soluble vascular cell adhesion molecule-1 levels in preeclampsia. Am J Reprod Immunol 41, 407–412 10392229 10.1111/j.1600-0897.1999.tb00455.x

[15] Austgulen R, Lien E, Vince G, et al. (1997) Increased maternal plasma levels of soluble adhesion molecules (ICAM-1, VCAM-1, E-selectin) in preeclampsia. Eur J Obstet Gynecol Reprod Biol 71, 53–58 9031960 10.1016/s0301-2115(96)02647-4

[16] Arteel GE, Briviba K & Sies H (1999) Protection against peroxynitrite. FEBS Lett 445, 226–230 10094462 10.1016/s0014-5793(99)00073-3

[17] Myatt L, Rosenfield RB, Eis AL, et al. (1996) Nitrotyrosine residues in placenta. Evidence of peroxynitrite formation and action. Hypertension 28, 488–493 8794838 10.1161/01.hyp.28.3.488

[18] Roggensack AM, Zhang Y & Davidge ST (1999) Evidence for peroxynitrite formation in the vasculature of women with preeclampsia. Hypertension 33, 83–89 9931086 10.1161/01.hyp.33.1.83

[19] Cockell AP, Learmont JG, Smárason AK, et al. (1997) Human placental syncytiotrophoblast microvillous membranes impair maternal vascular endothelial function. Br J Obstet Gynaecol 104, 235–240 9070146 10.1111/j.1471-0528.1997.tb11052.x

[20] Goswami D, Tannetta DS, Magee LA, et al. (2006) Excess syncytiotrophoblast microparticle shedding is a feature of early-onset pre-eclampsia, but not normotensive intrauterine growth restriction. Placenta 27, 56–61 16310038 10.1016/j.placenta.2004.11.007

[21] Redman CW, Sacks GP & Sargent IL (1999) Preeclampsia: an excessive maternal inflammatory response to pregnancy. Am J Obstet Gynecol 180, 499–506 9988826 10.1016/s0002-9378(99)70239-5

[22] Roberts JM, Taylor RN, Musci TJ, et al. (1989) Preeclampsia: an endothelial cell disorder. Am J Obstet Gynecol 161, 1200–1204 2589440 10.1016/0002-9378(89)90665-0

[23] Rayman MP (2011) Selenium and adverse conditions of human pregnancy. In Selenium: Its Molecular Biology and Role in Human Health, 3rd ed., pp. 531–546 [Hatfield DL, Berry MJ and Gladyshev VN, editors]. New York: Springer Science+Business Media, LLC

[24] Rayman MP (2012) Selenium and human health. Lancet 379, 1256–1268 22381456 10.1016/S0140-6736(11)61452-9

[25] Brigelius-Flohé R, Banning A & Schnurr K (2003) Selenium-dependent enzymes in endothelial cell function. Antioxid Redox Signal 5, 205–215 12716480 10.1089/152308603764816569

[26] Lewin MH, Arthur JR, Riemersma RA, et al. (2002) Selenium supplementation acting through the induction of thioredoxin reductase and glutathione peroxidase protects the human endothelial cell line EAhy926 from damage by lipid hydroperoxides. Biochim Biophys Acta 1593, 85–92 12431787 10.1016/s0167-4889(02)00333-6

[27] Gao Y, Hannan NR, Wanyonyi S, et al. (2006) Activation of the selenoprotein SEPS1 gene expression by pro-inflammatory cytokines in HepG2 cells. Cytokine 33, 246–251 16574427 10.1016/j.cyto.2006.02.005

[28] Ejima K, Layne MD, Carvajal IM, et al. (2002) Modulation of the thioredoxin system during inflammatory responses and its effect on heme oxygenase-1 expression. Antioxid Redox Signal 4, 569–575 12230868 10.1089/15230860260220067

[29] Trigona WL, Mullarky IK, Cao Y, et al. (2006) Thioredoxin reductase regulates the induction of haem oxygenase-1 expression in aortic endothelial cells. Biochem J 394, 207–216 16209660 10.1042/BJ20050712 PMC1386018

[30] Vanderlelie JJ & Perkins AV (2011) Selenium and preeclampsia: a global perspective. Pregnancy Hypertens 1, 213–224 10.1016/j.preghy.2011.07.001 26009029

[31] Han L & Zhou SM (1994) Selenium supplement in the prevention of pregnancy induced hypertension. Chin Med J (Engl) 107, 870–871 7867399

[32] Atamer Y, Koçyigit Y, Yokus B, et al. (2005) Lipid peroxidation, antioxidant defense, status of trace metals and leptin levels in preeclampsia. Eur J Obstet Gynecol Reprod Biol 119, 60–66 15734086 10.1016/j.ejogrb.2004.06.033

[33] Chamy VM, Lepe J, Catalán A, et al. (2006) Oxidative stress is closely related to clinical severity of pre-eclampsia. Biol Res 39, 229–236 16874398 10.4067/s0716-97602006000200005

[34] Mistry HD, Wilson V, Ramsay MM, et al. (2008) Reduced selenium concentrations and glutathione peroxidase activity in preeclamptic pregnancies. Hypertension 52, 881–888 18852388 10.1161/HYPERTENSIONAHA.108.116103

[35] Maleki A, Fard MK, Zadeh DH, et al. (2011) The relationship between plasma level of Se and preeclampsia. Hypertens Pregnancy 30, 180–187 20818958 10.3109/10641950903322931

[36] Ghaemi SZ, Forouhari S, Dabbaghmanesh MH, et al. (2013) A prospective study of selenium concentration and risk of preeclampsia in pregnant Iranian women: a nested case–control study. Biol Trace Elem Res 152, 174–179 23354545 10.1007/s12011-013-9614-y

[37] Walsh SW & Wang Y (1993) Deficient glutathione peroxidase activity in preeclampsia is associated with increased placental production of thromboxane and lipid peroxides. Am J Obstet Gynecol 169, 1456–1461 8267046 10.1016/0002-9378(93)90418-i

[38] Moses EK, Johnson MP, Tømmerdal L, et al. (2008) Genetic association of preeclampsia to the inflammatory response gene SEPS1. Am J Obstet Gynecol 198, 336e1–336e5 18068137 10.1016/j.ajog.2007.09.024

[39] Vanderlelie J, Venardos K & Perkins AV (2004) Selenium deficiency as a model of experimental pre-eclampsia in rats. Reproduction 128, 635–641 15509710 10.1530/rep.1.00260

[40] Rayman MP, Abou-Shakra FR, Ward NI, et al. (1996) Comparison of selenium levels in pre-eclamptic and normal pregnancies. Biol Trace Elem Res 55, 9–20 8971350 10.1007/BF02784164

[41] Rayman MP, Bode P & Redman CWG (2003) Low selenium status is associated with the occurrence of the pregnancy disease preeclampsia in women from the United Kingdom. Am J Obstet Gynecol 189, 1343–1349 14634566 10.1067/s0002-9378(03)00723-3

[42] Department of Health (1991) Dietary reference values for food, energy and nutrients for the United Kingdom. Report of the Panel on Dietary Reference Values of the Committee on Medical Aspects of Food Policy (COMA). Rep Health Soc Subj (Lond) 41, 1–210 1961974

[43] Tidwell SC, Ho HN, Chiu WH, et al. (2001) Low maternal serum levels of placenta growth factor as an antecedent of clinical preeclampsia. Am J Obstet Gynecol 184, 1267–1272 11349200 10.1067/mob.2001.113129

[44] Levine RJ, Lam C, Qian C, et al. (2006) Soluble endoglin and other circulating antiangiogenic factors in preeclampsia. N Engl J Med 355, 992–1005 16957146 10.1056/NEJMoa055352

[45] Muttukrishna S, North RA, Morris J, et al. (2000) Serum inhibin A and activin A are elevated prior to the onset of pre-eclampsia. Hum Reprod 15, 1640–1645 10875882 10.1093/humrep/15.7.1640

[46] Anim-Nyame N, Gamble J, Sooranna SR, et al. (2003) Evidence of impaired microvascular function in pre-eclampsia: a non-invasive study. Clin Sci (Lond) 104, 405–412 12653685 10.1042/

[47] García RG, Celedón J, Sierra-Laguado J, et al. (2007) Raised C-reactive protein and impaired flow-mediated vasodilation precede the development of preeclampsia. Am J Hypertens 20, 98–103 17198919 10.1016/j.amjhyper.2006.06.001

[48] Dubin R, Li Y, Ix JH, et al. (2012) Associations of pentraxin-3 with cardiovascular events, incident heart failure, and mortality among persons with coronary heart disease: data from the Heart and Soul Study. Am Heart J 163, 274–279 22305847 10.1016/j.ahj.2011.11.007 PMC3273726

[49] Burk RF & Hill KE (2009) Selenoprotein P – expression, functions, and roles in mammals. Biochim Biophys Acta 1790, 1441–1447 19345254 10.1016/j.bbagen.2009.03.026 PMC2763998

[50] Sloth JJ & Larsen EH (2000) The application of ICP dynamic reaction cell mass spectrometry for measurement of selenium isotopes, isotope ratios and chromatographic detection of selenoamino acids. J Anal Atom Spectrom 15, 669–672

[51] Sieniawska C, Meniskov R & Delves HT (1999) Determination of total selenium in serum, whole blood and erythrocytes by ICP-MS. J Anal Atom Spectrom 14, 109–112

[52] Burk RF, Norsworthy BK, Hill KE, et al. (2006) Effects of chemical form of selenium on plasma biomarkers in a high-dose human supplementation trial. Cancer Epidemiol Biomarkers Prev 15, 804–810 16614127 10.1158/1055-9965.EPI-05-0950

[53] Tannetta DS, Muttukrishna S, Groome NP, et al. (2003) Endothelial cells and peripheral blood mononuclear cells are a potential source of extraplacental activin A in preeclampsia. J Clin Endocrinol Metab 88, 5995–6001 14671202 10.1210/jc.2002-021924

[54] Gillespie SH, Dow C, Raynes JG, et al. (1991) Measurement of acute phase proteins for assessing severity of *Plasmodium falciparum* malaria. J Clin Pathol 44, 228–231 1707416 10.1136/jcp.44.3.228 PMC496944

[55] Ross MG & Idah R (2004) Correlation of maternal plasma volume and composition with amniotic fluid index in normal human pregnancy. J Matern Fetal Neonatal Med 15, 104–108 15209117 10.1080/14767050310001650770

[56] Behne D, Alber D & Kyriakopoulos A (2010) Long-term selenium supplementation of humans: selenium status and relationships between selenium concentrations in skeletal muscle and indicator materials. J Trace Elem Med Biol 24, 99–105 20413067 10.1016/j.jtemb.2009.12.001

[57] Whittaker PG & Lind T (1993) The intravascular mass of albumin during human pregnancy: a serial study in normal and diabetic women. Br J Obstet Gynaecol 100, 587–592 8334096 10.1111/j.1471-0528.1993.tb15315.x

[58] Davey DA & MacGillivray I (1988) The classification and definition of the hypertensive disorders of pregnancy. Am J Obstet Gynecol 158, 892–898 3364501 10.1016/0002-9378(88)90090-7

[59] Rana S, Powe CE, Salahuddin S, et al. (2012) Angiogenic factors and the risk of adverse outcomes in women with suspected preeclampsia. Circulation 125, 911–919 22261192 10.1161/CIRCULATIONAHA.111.054361 PMC3319742

[60] Saudan P, Brown MA, Buddle ML, et al. (1998) Does gestational hypertension become pre-eclampsia? Br J Obstet Gynaecol 105, 1177–1184 9853766 10.1111/j.1471-0528.1998.tb09971.x

[61] Brown MA & de Swiet M (1999) Classification of hypertension in pregnancy. Baillieres Best Pract Res Clin Obstet Gynaecol 13, 27–39 10746091 10.1053/beog.1999.0004

[62] Barton JR, O'Brien JM, Bergauer NK, et al. (2001) Mild gestational hypertension remote from term: progression and outcome. Am J Obstet Gynecol 184, 979–983 11303208 10.1067/mob.2001.112905

[63] Davis GK, Mackenzie C, Brown MA, et al. (2007) Predicting transformation from gestational hypertension to preeclampsia in clinical practice: a possible role for 24 hour ambulatory blood pressure monitoring. Hypertens Pregnancy 26, 77–87 17454220 10.1080/10641950601147952

[64] Butler JA, Whanger PD & Tripp MJ (1982) Blood selenium and glutathione peroxidase activity in pregnant women: comparative assays in primates and other animals. Am J Clin Nutr 36, 15–23 7091025 10.1093/ajcn/36.1.15

[65] Zachara BA, Wardak C, Didkowski W, et al. (1993) Changes in blood selenium and glutathione concentrations and glutathione peroxidase activity in human pregnancy. Gynecol Obstet Invest 35, 12–17 8449427 10.1159/000292655

[66] Burk RF, Olson GE, Hill KE, et al. (2013) Maternal–fetal transfer of selenium in the mouse. FASEB J 27, 3249–3256 23651543 10.1096/fj.13-231852 PMC3714584

[67] Vunta H, Davis F, Palempalli UD, et al. (2007) The anti-inflammatory effects of selenium are mediated through 15-deoxy-Δ12,14-prostaglandin J2 in macrophages. J Biol Chem 282, 17964–17973 17439952 10.1074/jbc.M703075200

[68] Vunta H, Belda BJ, Arner RJ, et al. (2008) Selenium attenuates pro-inflammatory gene expression in macrophages. Mol Nutr Food Res 52, 1316–1323 18481333 10.1002/mnfr.200700346

[69] Bainbridge SA & Smith GN (2005) HO in pregnancy. Free Radic Biol Med 38, 979–988 15780756 10.1016/j.freeradbiomed.2004.11.002

[70] Cudmore M, Ahmad S, Al-Ani B, et al. (2007) Negative regulation of soluble Flt-1 and soluble endoglin release by heme oxygenase-1. Circulation 115, 1789–1797 17389265 10.1161/CIRCULATIONAHA.106.660134

[71] Kirkby KA & Adin CA (2006) Products of heme oxygenase and their potential therapeutic applications. Am J Physiol Renal Physiol 290, F563–F571 16461755 10.1152/ajprenal.00220.2005

[72] Ahmed A, Rahman M, Zhang X, et al. (2000) Induction of placental heme oxygenase-1 is protective against TNFalpha-induced cytotoxicity and promotes vessel relaxation. Mol Med 6, 391–409 10952020 PMC1949957

[73] Traulsen H, Steinbrenner H, Buchczyk DP, et al. (2004) Selenoprotein P protects low-density lipoprotein against oxidation. Free Radic Res 38, 123–128 15104205 10.1080/10715760320001634852

[74] Thomson CD, Robinson MF, Butler JA, et al. (1993) Long-term supplementation with selenate and selenomethionine: selenium and glutathione peroxidase (EC 1.11.1.9) in blood components of New Zealand women. Br J Nutr 69, 577–588 8490010 10.1079/bjn19930057

[75] Hurst R, Armah CN, Dainty JR, et al. (2010) Establishing optimal selenium status: results of a randomized, double-blind, placebo-controlled trial. Am J Clin Nutr 91, 923–931 20181815 10.3945/ajcn.2009.28169 PMC2844680

[76] Kimmons JE, Blanck HM, Tohill BC, et al. (2006) Associations between body mass index and the prevalence of low micronutrient levels among US adults. MedGenMed 8, 59 17415336 PMC1868363

[77] Arnaud J, Bertrais S, Roussel AM, et al. (2006) Serum selenium determinants in French adults: the SU.VI.M.AX study. Br J Nutr 95, 313–320 16469147 10.1079/bjn20051528

[78] Méplan C, Crosley LK, Nicol F, et al. (2007) Genetic polymorphisms in the human selenoprotein P gene determine the response of selenoprotein markers to selenium supplementation in a gender-specific manner (the SELGEN study). FASEB J 21, 3063–3074 17536041 10.1096/fj.07-8166com

[79] Bodnar LM, Tang G, Ness RB, et al. (2006) Periconceptional multivitamin use reduces the risk of preeclampsia. Am J Epidemiol 164, 470–477 16772374 10.1093/aje/kwj218

[80] Rayman MP (2008) Food-chain selenium and human health: emphasis on intake. Br J Nutr 100, 254–268 18346308 10.1017/S0007114508939830

